# The relationship between bone health and plasma zinc, copper lead and cadmium concentration in osteoporotic women

**DOI:** 10.1186/s40201-014-0125-3

**Published:** 2014-11-19

**Authors:** Naficeh Sadeghi, Mohammad Reza Oveisi, Behrooz Jannat, Mannan Hajimahmoodi, Masoomeh Behzad, Abdolazim Behfar, Fatemeh Sadeghi, Sahereh Saadatmand

**Affiliations:** Department of Drug and Food Control, School of Pharmacy, Tehran university of Medical Sciences, Tehran, Iran; Halal research center, Ministry of Health and Medical Education, Tehran, Iran; Department of Drug and Food Control, Faculty of Pharmacy, Jondishapour Ahvaz University of Medical Sciences, Ahvaz, Iran

**Keywords:** Zinc, Copper, Lead, Cadmium, Osteoporosis, Voltammetry

## Abstract

Osteoporosis is a multi factorial disease with dimension of genetic and nutritional considerations. The aim of this study was to present data from the association of plasma zinc, copper and toxic elements of lead and cadmium levels with bone mineral density in Iranian women. 135 women gave their information and enrolled. Fasting plasma was used for measurement of trace elements and heavy metals by Differential Pulse Anodic Stripping Voltammetry. Control group (n = 51) were normal in both lumbar spine (L_1_-L_4_) and femoral neck density (T-score ≥ −1), but just femoral neck T-score was considered as criterion in selection of patient group (n = 49, Tscore < −1). No differences were found in the nutritional status, number of diseases, drugs and functional activities between these groups. Plasma Zn, Cu, Pb, Cd levels were analyzed by, a method of voltammetry. Mean ± SD levels of copper and zinc was 1.168 ± 0.115, 1.097 ± 0.091 μg/ml in control group, 1.394 ± 0.133, 1.266 ± 0.11 μg/ml in total patient (TP) and 1.237 ± 0.182, 1.127 ± 0.176 μg/ml in Mild patients(−1 > T-score > −1.7), 1.463 ± 0.174, 1.327 ± 0.147 μg/ml in Severe patient group (T-score < −1.7); respectively. Mean ± SD plasma level of lead and cadmium was 168.42 ± 9.61 ng/l, 2.91 ± 0.18 ng/ml in control group, 176.13 ± 8.64 ng/l, 2.97 ± 0.21 ng/ml in TP, 176.43 ± 13.2 ng/l, 2.99 ± 0.1 ng/ml in mild patients, 221.44 ± 20 ng/l and 3.80 ± 0.70 ng/ml in severe patient group, respectively. In this study plasma zinc, copper, lead & cadmium concentrations were higher in the patients than in the control, though differences were not significant. However, differences were higher between the controls and patients with severe disease (T-score < −1.7). In addition adjusted T-score of femur with age and BMI showed negative significant correlation with plasma levels of zinc and lead in total participants (*p* < 0.05, r = −0.201, *p* = 0.044, r = −0.201). It seems that more extensive study with larger ample size might supply definite results about this association for copper and cadmium.

## Background

Osteoporosis is a skeletal disorder characterized by compromised bone strength predisposing to an increased risk of fracture [1,2]. The introduction of bone density measurement methods makes it possible to diagnose osteoporosis before fractures occur. The World Health Organization (WHO) has defined osteoporosis as a bone mineral density (BMD) at the hip or spine that is ≤ −2.5 SDs (standard deviation) below the mean value for young women, and osteopenia was defined as a BMD that is between −1 and −2.5 SDs below this value [3]. Zinc is essential element for growth of human. Role of zinc is demonstrated in the growth and mineralization and preservation of bone tissue. Copper has an essential role in the normal maturation of collagen, particularly in the important steps to the formation of lysine- derived cross-link [4,5]. Lead is a highly toxic metal that may damage directly bone-forming cells (increasing chondrogenesis, delaying cartilage mineralization) and alter the hormonal regulation of Calcium and vitamin D3. Lead has a negative effect on the processes regulating bone turnover mechanisms and bone maturation or skeletal growth. These changes may affect bone fragility and the risk of fractures [6]. Lead will be retained in bone by the replacement of calcium. Cadmium is a widespread environmental pollutant, present in food and tobacco. Cadmium may have both direct and indirect effects on bone turnover [7,8]. Stripping voltammetry (SV) analysis is powerful and simple tool to determination of trace elements. In trace analysis of metal ions, Anodic stripping voltammetry (ASV) is the most popular SV technique [9]. The aim of this study is determination of plasma levels of lead, cadmium, zinc and copper in osteoporotic women comparison to control by voltammetric method and how environmental toxins, lead and cadmium leads to the pathogenesis of osteoporosis.

## Methods

### Apparatus

The polarograph set was Trace analyzer model 746 instruments (Metrohm Ltd. Switzerland). Hanging Mercury Drop Electrode (HMDE), Reference silver chloride electrode (Ag/Agcl/kcl 3 M) and platinum (Pt) auxiliary electrode were used.

### Reagents

The chemical, CH_3_COONa.3H_2_O, Pb(NO_3_)_2_, Cd(NO_3_)_2_, Cu(NO_3_)_2_, Zn(NO_3_)_2_ and HNO_3_ used in analytical grades and were purchased from Merck Company Germany. Stock solution (1 g/L) of 0.1 Molar nitric acid was prepared. Hydrochloric acid (HCL) used to acidity the plasma samples was Suprapur grade of Merck Company. The stock solution (1 g/L) was prepared in 2.5 normal HCL. The pH acetate buffer was adjusted between 4.6 and 4.8.

### Sample preparation

In the present study, people were screened through a total of approximately 1000 women that referred to bone mineral densitometry division of Jami Clinic in Tehran, Iran. 135 women gave their information and enrolled. Participants were separated into three groups according to T-score values suggested by WHO. The control group T-score is ≥ −1 and the total patients (mild osteopenia + severe osteopenia + osteoporosis) T-score < −1. The participants underwent a fasting blood withdrawal in 10-ml heparinized tubes on the day of the bone scan and then plasma was seprated. A number of 200 μL of plasma was added to 800 μL of 2.5 N hydrochloric acid to percipitate plasma proteins and centrifuged for 15 min at 6000 rpm. Then the clear supernatant passed from 0.2 micron filter before transferring to the polarographic cell. The complex composition of the plasma may cause changes in the calibration slopes; that is why, the zinc, copper, lead & cadmium concentrations were determined using the standard addition technique with three additions of standard stock solution. First 10 ml of 0.1 M acetate buffer was used to set the blank polarogram. Next 500 μL of sample solution was added to polarographic cell and the polarogram was plotted. After that 100 μL of the standard stock solution was added to the cell for three times and each time the polarogram was done. The cell solution was deoxygenated by nitrogen for 300 seconds. Determination was made on a Hanging mercury drop electrode (HMDE). Determination of zinc and copper was made after a collection period at a potential of −1200 and −500 mV respectively. Method was able to give estimation of cadmium and lead simultaneously when a sweep potential was applied between −800 and −100 mV. The potential was swept, using a differential pulse anodic stripping voltammetry (DPASV) [9,10]. Three determinations were done for each sample, and the standard deviations were calculated [10].

### Ethical approval

The project was approved by Ethics Committee of Tehran University of Medical Sciences (reference number of approval 89-01-33-10034).

### Consent

We obtained in formed consent from all participants but no patient was reported as a case.

## Results and discussion

In the current study the level of zinc, copper, lead and cadmium were determined in plasma of women with osteoporosis. The total number of subjects was 135. The T- score of both lumbar spine (L1-L4) and femoral neck were measured in all participants. The control group was normal in both femur and spine with T-score ≥ −1 including 51 women (37.8%). The total patient (mild osteopenia + severe osteopenia + osteoporosis) had femoral T-score < −1 including 49 women (36.3%) and women with −1.7 ≤ T-score < −1 as mild osteopenia, patients with −2.5 < T-score < −1.7 as severe osteopenia including 41 women (30.3%). The total number of samples from 35 (25.9%) were not including in the classification of cases and controls; because of femoral T-score > −1 but lumbar T-score < −1, so they were analyzed in total participants data. No difference in menopause, drugs, nutritional habit, functional activities, stress and exposure time in the sun was found between groups, but the difference was significant for smoking (*P* < 0.05), age and BMI (*P* < 0.01) (Table 1). The concentrations of zinc, copper, lead and cadmium in each patient division were compared with control. The regression relationship between the amount of zinc, copper, lead and cadmium with femoral T-score in control and patient groups were compared. No significant relationship was detected between age and BMI with the concentration of zinc, copper, lead and cadmium. Zinc, copper, lead and cadmium levels are higher in smokers than non-smokers, but the difference is not significant (*p* = 0.266, *p* = 0.150, *p* = 0.146, *p* = 0.462 respectively). Results are shown in Table 2.

**Table 1:** **Comparison of age and BMI among control and patient groups**

**Group**	**Control**	**Total patients**
	**T-score > −1**	**T-score ≤ −1**
	**(Mean ± SD)**	
	n = 51	n = 49
**Age (year)**	48.17 ± 11.16	55.8 ± 12.38^α^
**BMI (Kg/m2)**	28.25 ± 50.02	25.8 ± 12.38^α^

^α^ *p* <0.01.

**Table 2:** **Femoral neck T-score and plasma concentrations of Zn, Cu, Pb, Cd in smokers and non-smokers**

	**Smokers**	**Non-smokers**
	**(Mean ± SD)**	
**Participants (%)**	12	88
**Average T-score femur**	−1.49 ± 0.99	0.62 ± 1.3
**Zinc (ng/ml)**	1512 ± 258	1108 ± 59
**Copper (ng/ml)**	1572 ± 282	1290 ± 79
**Lead (ng/l)**	196.71 ± 20.93	169.47 ± 5.81
**Cadmium (ng/ml)**	2.98 ± 0.12	2.69 ± 0.42

Also exercise and inactivity, menopause and pre-menopausal status showed no significant effect on zinc, copper, lead and cadmium levels. An inverse relationship between age and femoral T-score is illustrated. Results are shown in Table 3.

**Table 3:** **Concentration of Zn, Cu, Pb, Cd in postmenopausal, Pre-menopausal, athlete and non-athlete participants**

	**Athlete**	**Non-athlete**	**Pre-menopausal**	**Post-menopausal**
		**(Mean ± SD)**		
**Zinc (μg /ml)**	1.079 ± 0.138	1.171 ± 0.067	1.138 ± 0.080	1.163 ± 0.090
**Copper (μg /ml)**	1.107 ± 0.155	1.369 ± 0.088	1.233 ± 0.105	1.428 ± 0.110
**Lead (ng/l)**	166.63 ± 11.82	173.54 ± 6.51	174.66 ± 8.53	169.11 ± 6.91
**Cadmium (ng/ml)**	2.74 ± 0.16	3.09 ± 0.14	2.87 ± 0.16	3.05 ± 0.16

Morefurther, a direct and significant relationship between BMI and T-score of femur was detected. In this study, plasma mean levels of zinc, copper, lead and cadmium were obtained for 135 samples. Results are shown in Table 4.

**Table 4:** **Plasma Mean ± SD levels of Zn, Cu, Pb, Cd in different groups**

	**T-score > −1**	**T-score ≤ −1**	**−1.7 < T-score < −1**	**−2.5 < T-score < −1.7**	**T-score < −2.5**
**Zn (μg/ml)**	1.1 ± 1.09	1.26 ± 0.11	1.13 ± 0.17	1.33 ± 0.15	1.27 ± 0.42
**Cu (μg/ml)**	1.17 ± 0.11	1.39 ± 0.13	1.24 ± 0.18	1.46 ± 0.17	1.71 ± 0.46
**Pb (ng/l)**	168.42 ± 0.01	176.13 ± 0.26	176.43 ± 13.2	183.06 ± 10.91	221.4 ± 20
**Cd (ng/ml)**	2.91 ± 0.18	2.97 ± 0.21	2.99 ± 0.19	3.13 ± 0.29	3.80 ± 0.70

The relationship between the concentrations of zinc, copper, lead and cadmium were studied with the T-score of femur Table 5.

**Table 5:** **Correlation between plasma concentrations of Zn, Cu, Pb, Cd and femoral T-score in different groups without age and BMI matching**

	**T-score < 2.5**	**T-score > −1**	**T-score ≤ −1**	**−1.7 < T-score < −1**	**−2.5 < T-score < −1.7**	**T-score < −2.5**
**Zn**	p: 0.041^**α**^	0.125	0.356	0.736	0.669	0.799
	r: −0.181	−0.218	−0.135	−0.095	−0.076	−0.135
**Cu**	p: 0.205	0.980	0.278	0.202	0.610	0.899
	r: −0.113	−0.004	−0.188	−0.349	−0.091	−0.067
**Pb**	p: 0.072	0.179	0.093	0.230	0.363	0.933
	r: −0.160	−0.191	−0.243	−0.330	−0.161	−0.045
**Cd**	p: 0.596	0.781	0.160	0.652	0.440	0.994
	r: −0.047	−0.040	−0.204	−0.127	−0.137	−0.004

^α^ *p* <0.05.

For total participant’s of135 after matching with age and BMI, plasma concentrations of Zn and Pb have a statistically significant negative relationship with T-score of femur. But the relationship between plasma concentrations of Cu and Cd with femoral T-score can be observed probably after sample size increasing. The results are shown in Table 6.

**Table 6:** **Correlation between concentrations of Zn, Cu, Pb and Cd with femoral T-score in total population after age and BMI matching**

	**Zn**	**Cu**	**Pb**	**Cd**
**Total participants**	p: 0.030^α^	0.063	0.044^α^	0.306
**(T-score <2.5)**	r: −0.216	−0.186	−0.201	−0.103

^α^ *p* <0.05.

In this study, plasma zinc, copper, lead & cadmium concentration among Iranian osteoporotic women showed that: plasma zinc, copper, lead & cadmium concentration were higher in the patients than in the control, though differences were not significant. However, differences were higher between the controls and patients with severe disease (T-score < −1.7). In addition, T-score of femur adjusted with interfere factors of age and BMI, showed negative significant correlation with plasma levels of Zn and Pb in the entire study population (*p* < 0.05, r = −0.201, *p* = 0.044, r = −0.201 respectively). It seems that more extensive study with larger ample size might supply definite results about this reverse linear association for copper and cadmium. Bone zinc content is decreased by development of aging, bone loss, and post menopausal conditions. The metal directly activates Aminoacyl-tRNA synthetase in osteoblastic cells and it stimulates cellular protein synthesis. Zinc may act on the process of bone-resorbing factors induced protein kinase C activation, which is involved in Ca^+2^ signaling in osteoclastic cells [11]. Zinc is an essential trace element that is a cofactor of more than 200 enzymes [12]. A decrease in the formation of cross-linking amino acids is thought account in part for the increased fragility of bone from copper-deficient [13]. Cadmium can interfere with vitamin D metabolism [7]. For many years, it has been thought that Cd affects bone only at high-level long-term exposure. Recent epidemiological data indicate that Cd can damage the skeleton at considerably lower exposure than previously anticipated; however, the critical level of the exposure is still unknown. Decreased bone density with increased risk of fractures has been reported in conditions of low to moderate environmental exposure to Cd taking place in industrialized countries. Until now, various mechanisms for the Cd-induced bone damage involving both direct and indirect action of this metal have been suggested, they might explainthe indirect mechanism involves the Cd-induced disorders in the metabolism of vitamin D and minerals due to kidney and gastrointestinal tract damage [14].

In 2009 Smith *et al*. [15] reported that cadmium causes apoptosis in human osteoblast-like Saos-2 cells. They showed that cadmium exposure induces oxidative stress which leads to decreased RUNX2 mRNA expression and increased apoptotic death, antioxidant NAC alleviates the damaging effects of cadmium. Furthermore the osteoblast transcriptional factor RUNX2 is reported to play a protective role against osteoporosis in postmenopausal women. Cadmium can indirectly induce oxidative stress through depletion of antioxidant molecules or inhibition of antioxidant enzymes. Mounting world-wide epidemiological research indicates that chronic, low level exposure to lead and cadmium leads to increased risk of bone fractures. The importance of the age factor in bone density is obvious and known to increase the risk of osteoporosis and bone density decreases [16]. The analysis of the results of this study indicated that the plasma concentrations of zinc, copper, lead and cadmium are not affected by age. Plasma zinc and copper concentrations were higher in those who did not exercise. Physical activity has a negative association with plasma concentrations of lead, though it is not significant. However, the plasma concentration of cadmium was lower in athletes, and this difference was significant (*p* < 0.01). In 2011 Margaret E. Sears [17] studied the amount of lead, cadmium, mercury and arsenic in sweat, so it was seen as much more sweat as more excretion of these elements. In other words more exercise means more perspiration and excretion of these elements, so plasma levels of these harmful elements reduce. Smoking is known as a cause of bone mineral loss and may increase the risk of hip fracture [18]. Polidori reports that α, γ tocopherol concentrations, α, β cryptoxanthin, retinol and ascorbate to increases after 4 weeks smoking, significantly. In the present study, we also observed an increase in plasma antioxidant capacity in smokers, though is not significant [19]. Kido *et al*. [20] study has shown an association between renal tubular dysfunction and decreased BMD in examination of women environmentally exposed to cadmium. A study in 1988 by Wittmers *et al*. [21] was performed for survey the effects of lead injection in bone and skeletal tissue. It was seen that Lead makes some spaces in bone. Lead cause snaping of Hydroxy apatit crystals in calcification process. This toxic substance remains in bone until remodeling. In a clinical study, Saltman [22] has demonstrated the efficacy of Ca, Cu, Mn and Zn supplementation in spinal bone mineral density in postmenopausal women and the necessity of trace elements for optimal bone matrix development and bone density sustenance. Yamaguchi *et al*. [23] investigated the effects of zinc on bone cells. Those cells that were exposed to more zinc concentration, significantly showed increased consumption of zinc, calcium, alkaline phosphatase and ATPase activity, also collagen was increased significantly. These findings suggest the direct effect of zinc on the mineralization of bone *in vitro* and stimulation of bone protein synthesis. Insufficient copper intake in the diet can cause osteoporosis, bone calcium and copper deficiency, bone abnormalities and curvature of the spine. Suttle [24] showed that bone formation activity is one of the first activities which are disrupted in copper-deficient lambs. Our results showed more zinc and copper levels in osteoporotic patients than controls, although not significantly. Some studies indicated that free radicals increase osteoporosis as well as the role of antioxidants in their lack of support, so the disease can progress. But it seems that the in oxidative stress disease, antioxidants have always decreased, although this may still be inadequate to meet the condition. Getting more antioxidants can help prevent the progression of osteoporosis.

## Conclusions

The comprehensive database shows that plasma levels of zinc, copper, lead and cadmium in the osteoporotic women is higher than control, though the differences were not significant. It seems that more extensive study with larger size might supply definite result about this association.
